# Predicting Syndromic Status Based on Longitudinal Data from Parental Reports of the Presence of Additional Structural and Functional Anomalies in Children Born with an Orofacial Cleft

**DOI:** 10.3390/jcm13226924

**Published:** 2024-11-17

**Authors:** Amy J. V. Davies, Yvonne E. Wren, Mark Hamilton, Jonathan R. Sandy, Evangelia Stergiakouli, Sarah J. Lewis

**Affiliations:** 1 The Cleft Collective, University of Bristol, Bristol BS8 2BN, UK; 2 School of Sport and Health Sciences, Cardiff Metropolitan University, Cardiff CF5 2YB, UK; 3 Bristol Speech and Language Therapy Research Unit, North Bristol NHS Trust, Bristol BS10 5NB, UK; 4 West of Scotland Regional Genetics Service, Laboratory Medicine Building, Queen Elizabeth University Hospital, Glasgow G51 4TF, UK; 5 Population Health Sciences, Bristol Medical School, University of Bristol, Bristol BS8 2BN, UK

**Keywords:** cleft lip, cleft palate, syndrome, the Cleft Collective, orofacial cleft, structural and functional anomalies

## Abstract

**Background:** Orofacial clefts are the most common craniofacial congenital malformation in humans. Approximately 30% of clefts arise as part of a syndrome or sequence, characterised by co-existing structural and functional anomalies. Many syndromes are thought to be undiagnosed, although the presence of multiple anomalies may indicate the presence of a syndrome or sequence. **Aim:** To determine the extent to which the presence of additional structural and functional anomalies can help to identify those children with an undiagnosed syndrome. **Methods:** Secondary data analysis was performed using data from 1701 children born with an orofacial cleft, collected as part of a longitudinal cohort study, the Cleft Collective. Data were collected between 2013 and 2023 across the United Kingdom. The prevalence of structural and functional anomalies and syndromes were explored using descriptive statistics. Logistic regression was used to determine the extent to which anomalies can predict syndromic status. **Results:** A syndrome and/or sequence was reported in 20.5% children. Among children who reported five or more anomalies, the prevalence of a diagnosed syndrome was 81.5%. When adjusting for cleft subtype and sex, in 27 out of 32 anomalies tested, strong evidence was found to suggest increased odds of having a syndrome if the specific anomaly was present compared to if the anomaly was absent (*p*-values ranged between 1.4 × 10^−30^ and 0.002). **Conclusions:** Children born with a cleft who present with two or more anomalies are much more likely to have a syndrome than those with fewer anomalies and should be prioritised for genetic screening and counselling.

## 1. Introduction

Orofacial clefts (OFCs) occur in approximately 1 in every 700 live births [1] and presents as one of five major subtypes: cleft lip (with or without alveolar involvement), cleft palate only, unilateral cleft lip and palate, bilateral cleft lip and palate and submucous cleft palate. Approximately 30% of OFCs are reported as part of a syndrome or sequence [2,3,4,5].

A syndrome is characterised by the presence of a recognisable pattern of co-existing structural and/or functional anomalies, which share a common underlying cause. Common anomalies associated with syndromic OFCs include additional craniofacial abnormalities, congenital heart anomalies, musculoskeletal malformations and developmental delay [6,7,8]. A sequence may also account for the presence of associated anomalies which have occurred as secondary effects of a single primary malformation [5]. An example includes Pierre Robin sequence where micrognathia during foetal development subsequently results in glossoptosis, failure of the palatine shelves to fuse and a restricted airway.

The aetiology of OFCs is complex and not yet fully understood [2]. However, it is known that syndromic OFCs are typically caused by a specific genetic abnormality or teratogenic exposure. Most non-syndromic OFCs are understood to have a complex basis, caused by a combination of genetic and environmental factors [2,9,10,11]. A child is more likely to be diagnosed with a syndrome or sequence if they were born with a cleft palate only compared to a child born with a cleft lip +/− palate [12,13].

In previous studies, the reported prevalence of co-occurring anomalies in children born with an OFC range between 2.9% and 39.5% [5,13,14,15,16,17,18,19,20,21,22,23]. The anomalies reported across these studies vary as does the geographical location. Factors contributing to differences in the ascertainment of syndromic cases likely include variable access and thresholds to undertake genetic investigations, as well as the duration of follow-up by the cleft service, since additional features that might suggest a syndromic cause may only emerge over time.

Within the United Kingdom of Great Britain and Northern Ireland (UK), children born with an OFC are managed within a multidisciplinary cleft team. While such teams generally have links with regional Clinical Genetics services or may include a named Clinical Geneticist, national consensus guidelines are presently lacking regarding which children born with an OFC should receive formal assessment by a Clinical Geneticist and/or be offered genetic tests. Consequently, there is likely to be considerable regional variation in practice. In this context, there is a pressing need for data to inform a rational approach to genetic investigation in this heterogeneous group of patients.

In addition, to enable robust analyses of genetic and environmental factors influencing OFCs, there is a need to stratify cohorts by syndromic status, i.e., whether the child has a syndrome or presents with features of a known sequence. This will reduce “noise” within the analysis as theoretically it will enable stratification between monogenic and multifactorial causation providing more power to detect an effect. Understanding the structural and functional anomalies occurring in children presenting with an apparently isolated OFC would also guide clinical management. For example, it could clarify where formal screening for associated malformations might be indicated or improve the accuracy of prognostic information available for children born with OFCs.

The aim of this study was to investigate the relationship between the prevalence, type and trajectory of structural and functional anomalies and syndromic diagnosis in children born with an OFC in the UK, considering differences across OFC subtypes and biological sex in order to characterise those children most at risk of having an undiagnosed syndrome.

### Objectives

The objectives of this study were as follows:

(1) Compare syndromic status among OFC subtypes and biological sex.
(2) Determine the extent to which the number of additional structural and functional anomalies present predicts the likelihood of a syndrome.
(3) Determine the prevalence of structural and functional anomalies in a cohort of children born with OFC.
(4) Determine the likelihood of a child having a syndrome when specific structural anomalies and functional deficits are present.
(5) Describe the presence of co-occurring structural and functional anomalies in children who were diagnosed with a syndrome present in five or more children.
(6) Explore the trajectory of syndromic diagnosis from the age of 18 months to 5 years.

## 2. Materials and Methods

### 2.1. Study Design and Setting

Secondary data analysis was performed using data from the Cleft Collective (project number CC038), a prospective longitudinal cohort study of children born with an OFC and their families [24,25,26]. Families were recruited to this study by one of the 16 cleft surgical centres across the UK. Recruitment to the Cleft Collective started in 2013 and is ongoing. Children born with an OFC and their families are recruited to either the birth cohort or the five-year-old cohort. Families within the birth cohort are either recruited during pregnancy (study child is not recruited until after birth) or after the birth of the study child but before the study child’s first surgical repair of their OFC. Families within the five-year cohort are recruited within the year proceeding the study child’s fifth birthday [26].

Parents of children in the birth cohort were asked to complete a baseline questionnaire at recruitment and follow-up questionnaires at key time points throughout their child’s development, including when their child reached the ages of 18 months, 3 years, 5 years, 8 years and 10 years. Parents of children in the five-year-old cohort were also asked to complete a baseline questionnaire at recruitment and follow-up questionnaires at key time points throughout their child’s development, including when their child reached the ages of 8 years and 10 years. Approximately 50% of baseline questionnaires and 38% of follow-up questionnaires were completed and returned. Parental questionnaires were available as paper versions and, since 2020, were also available digitally using REDCap (Research Electronic Data Capture) Nashville, TN, USA, version 14.5.6, hosted by the University of Bristol, Bristol, UK, and originally created by Vanderbilt University [27,28]. Further information on REDCap can be found here: https://projectredcap.org/ (accessed on 15 October 2024). Details on how to access the Cleft Collective resource can be found here: https://www.bristol.ac.uk/cleft-collective/professionals/access/ (accessed on 15 October 2024).

### 2.2. Structural and Functional Anomalies

Within the follow-up questionnaires, parents were asked if their child had any of the 32 anomalies listed (Supplementary File S1). In the general population of the UK, there are high prevalence rates of otitis media with effusion (OME), allergies, asthma and skin conditions among children. These conditions were therefore excluded from all analyses, although prevalence was reported for information within the results. To reduce the likelihood of double counting, structural and functional anomalies which could be considered similar were combined to form one group. Anomalies that we combined into individual groups were (1) “problems with the development of eyes” with “difficulties with vision/blindness”, (2) “problems with the development of ears” with “hearing loss or impairment”, (3) “problems with the development of feet” with “talipes” and (4) “problems with the development of spine” with “spine condition”.

Where a mother and father’s response differed, the anomaly was marked as being present. Where responses differed over time points, the latest response was used within the analysis. There was no clinical verification as to the diagnoses of structural and functional anomalies.

### 2.3. Syndromic Status

All follow-up questionnaires asked parents whether the study child has a syndrome and/or sequence, and if so, they were asked to specify which syndrome their child has (Supplementary File S1). There was no clinical verification of the parent-reported syndromic status.

### 2.4. Covariates

Additional variables included the child’s biological sex, OFC subtype and child’s age at the last completed parental follow-up questionnaire. Biological sex and OFC subtype were obtained through parental questionnaires and where possible were verified using data from other sources such as a clinical report of OFC subtype obtained from surgical questionnaires. OFC subtype was split into four categories: cleft lip only, cleft palate only, unilateral cleft lip and palate and bilateral cleft lip and palate. Children with a submucous cleft palate were excluded from the analysis due to the small numbers within our sample (n < 5). Date of completion and child’s date of birth were used to calculate child’s age in months at the time of completion of the latest questionnaire returned.

### 2.5. Statistical Analyses

Descriptive statistics were used to explore sample characteristics. The odds of having a syndrome by OFC subtypes and biological sex were estimated using logistic regression and binomial confidence intervals. Analyses for subtype were adjusted for biological sex and vice versa, due to OFC subtype being the strongest predictor of a syndrome/sequence and the prevalence of subtypes differing by sex.

A discrete variable was derived to determine the number of co-existing structural and functional anomalies for each child. The percentage of children with a syndromic diagnosis by the number of co-occurring structural and functional anomalies were described. The prevalence of individual structural and functional anomalies was described, with stratification by OFC subtype and biological sex. Furthermore, the prevalence of the 10 most commonly occurring combinations of structural and functional anomalies were described.

To identify the odds of having a diagnosed syndrome when individual structural and functional anomalies were present, a series of logistic regression models were performed; these models were adjusted for OFC subtype and biological sex and binomial confidence intervals were calculated. Since many anomalies were tested, a Bonferroni correction was applied. Due to the nature of Pierre Robin sequence presenting with multiple anomalies and the unlikely event that the presence of the sequence would be undiagnosed, isolated cases of Pierre Robin sequence were excluded from these analyses to ensure effect sizes were not inflated.

Where five or more children from the overall sample had been diagnosed with the same syndrome, the anomalies collectively present among this group of children were described. Where at least one parent had responded to all three of the 18-month, 3-year and the 5-year follow-up questionnaires, the prevalence of syndromic status and presence of two or more co-occurring anomalies between these ages were described.

All analyses were performed in Stata (version 18.0 MP, StataCorp LP, College Station, TX, USA).

### 2.6. Missing Data

Data were available for children within the Cleft Collective where at least one parent had responded to one follow-up questionnaire (46% of the cohort). Missing data within the questionnaires, for the questions of interest, were less than 5%. Multiple imputation was not performed for the remainder of the cohort as the covariates needed to impute the data were unknown. Currently, there is little evidence in the literature regarding the predictors for each of the individual structural and functional anomalies used within the analysis. Additional predictors for syndromic status would include genetic factors and a detailed family history of OFCs. Aside from co-occurring structural and functional anomalies, the strongest predictor for syndromic status within our dataset is OFC subtype. To identify whether our sample was representative of the distribution of OFC subtypes within the UK, we compared the distribution of our sample to the distribution of OFC subtypes seen within the Cleft Registry and Audit NEtwork (CRANE), UK.

### 2.7. Ethics Approval

Ethical approval for the Cleft Collective was obtained from South West Bristol Research Ethics Committee (13/SW/0064).

## 3. Results

**Figure 1:**
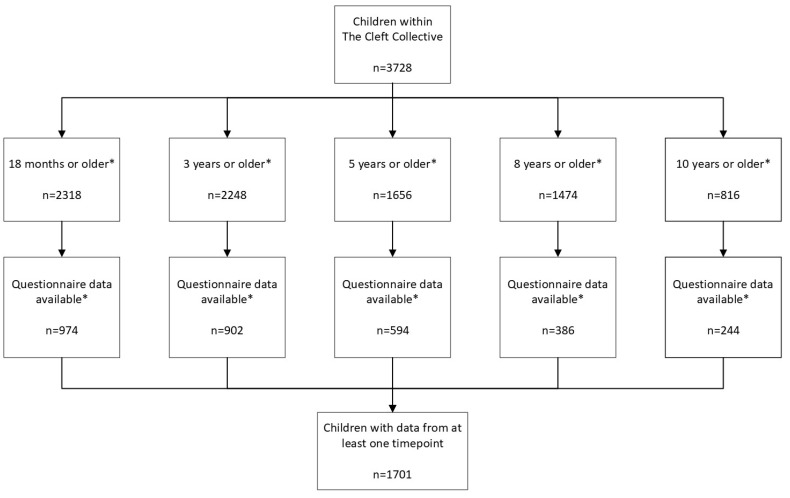
Flowchart of study children within the sample as of February 2023. * Figures reported are not mutually exclusive.

The sample comprised 1701 children born with an OFC who had data available on both syndromic status and structural and functional anomalies (Figure 1). Within the Cleft Collective sample, 24.9% (n = 423) were born with a cleft lip only, 38.4% (n = 653) were born with a cleft palate only, 26.2% (n = 446) were born with a unilateral cleft lip and palate, and 10.5% (n = 179) were born with a bilateral cleft lip and palate (Table 1). Comparison with the births of children born with an OFC in the UK and registered within CRANE between 2020 and 2022 are reported in Table S1 to demonstrate similarities between the distribution of the samples. The total sample comprised more males (57.8%, n = 983) than females (42.2%, n = 718) (Table 1). The presence of a syndrome and/or sequence was reported in 20.5% (n = 348) of our sample. Overall, 11.7% (n = 199) reported having Pierre Robin sequence and 22.0% (n = 44) of children with Pierre Robin sequence also had a syndrome. When excluding those children with isolated Pierre Robin sequence (n = 149) from the syndromic group, 11.3% (n = 193) of the sample had reported a diagnosed syndrome. A syndrome or sequence was diagnosed in 38.6% (n = 252) of children with a cleft palate, 17.9% (n = 32) of children with a bilateral cleft lip and palate, 8.1% (n = 36) of children with a unilateral cleft lip and palate and 6.6% (n = 28) of children with a cleft lip only (Figure S1). Strong evidence was found to suggest a syndrome and/or sequence was more likely to occur in children with a cleft palate only than with a cleft lip only (OR 8.55; 95% CIs 5.64, 12.96; *p* = 5.7 × 10^−24^) and more likely to occur in children with a bilateral cleft lip and palate than with a cleft lip only (OR 3.16; 95% CIs 1.83, 5.43; *p* = 3.3 × 10^−5^). The presence of a syndrome and/or sequence was similar between children with a cleft lip only (reference category) and with a unilateral cleft lip and palate (OR 1.26; 95% CIs 0.75, 2.11; *p* = 0.375). When adjusted for OFC subtype, weak evidence was found to suggest an association between syndrome and/or sequence diagnosis and biological sex (OR 1.25; 95% CIs 0.96, 1.62; *p* = 0.097) (Figure S2).

Among children who had no additional structural and functional anomalies, 8.1% (n = 62) were diagnosed with a syndrome or sequence. When excluding isolated Pierre Robin sequence, 3.8% (n = 28) of children who reported no additional anomalies had been diagnosed with a syndrome. The proportion of children with a diagnosed syndrome increased as the number of structural and functional anomalies also increased. For children who reported four structural and functional anomalies, 50.0% (n = 33) were diagnosed with a syndrome or sequence. When excluding isolated Pierre Robin sequence, 41.1% (n = 23) of children who reported four anomalies had been diagnosed with a syndrome (Table 2).

**Figure 2:**
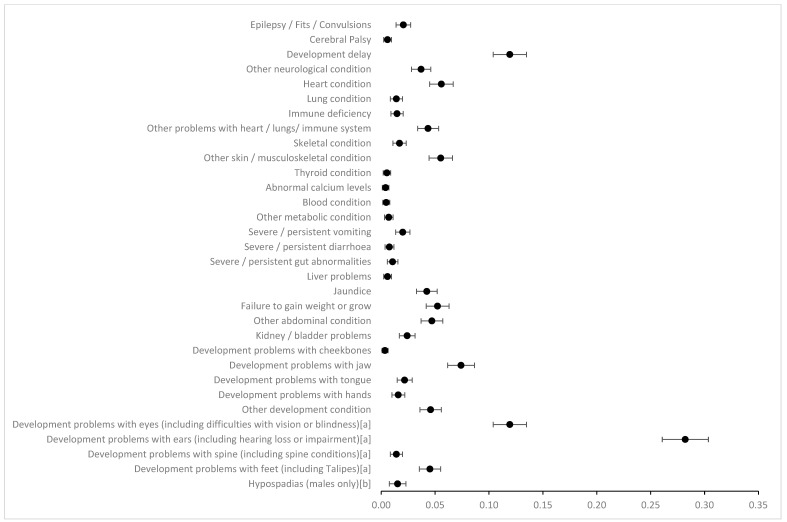
Prevalence of structural and functional anomalies with 95% confidence intervals. Overall sample, n = 1701. ^a^ Combined categories; ^b^ males only, n = 983.

The prevalence of OME, asthma, allergies and skin conditions was 46.3%, 11.1%, 9.8% and 12.3%, respectively, in our cohort, and as these are common childhood conditions in the UK, they were not included in our definition of additional anomalies. The anomaly with the highest prevalence included in further analysis was development problems with the ears (including hearing loss or impairment), recorded as 28.2%. The prevalence of development problems with the eyes (including difficulties with vision or blindness) and developmental delay were both recorded as 11.9% (Figure 2). The prevalence of all other anomalies was less than 10% (Table S2a). Prevalence of anomalies stratified by OFC subtype and biological sex are shown in Tables S2b and S2c, respectively.

When exploring the prevalence of structural and functional anomalies by syndromic status, the anomaly with the highest prevalence in children who had been diagnosed with a syndrome or a sequence was development problems with the ears (including hearing loss or impairment), recorded as 41.7%. Other anomalies which had a prevalence of over 25% for those who had been diagnosed with a syndrome or sequence were developmental delay (30.2%) and development problems with the jaw (27.3%). The anomaly with the highest prevalence in children who had been diagnosed with a syndrome excluding isolated Pierre Robin sequence was also development problems with the ears (including hearing loss or impairment), recorded as 43.0%. Other anomalies which had a prevalence of over 25% for those who had been diagnosed with a syndrome excluding isolated Pierre Robin sequence were developmental delay (41.5%) and development problems with the eyes (including difficulties with vision or blindness) (30.6%) (Table 3).

Within children identified as having two or more anomalies (n = 400), development problems with the ears and developmental delay commonly occurred alongside another anomaly. For children who were identified as having two or more anomalies, development problems with the ears were reported alongside developmental delay in 25.3% (n = 101), development problem with the eyes in 21.3% (n = 85), development problems with the jaw in 10.5% (n = 42), other skin and musculoskeletal conditions in 9.8% (n = 39), failure to gain weight or grow in 9.5% (n = 38), heart conditions in 9.0% (n = 36) and development problems with the feet in 8.3% (n = 33). Developmental delay was also frequently reported alongside anomalies other than developmental problems with the ears, including development problem with the eyes in 11.5% (n = 46), failure to gain weight or grow in 8.8% (n = 35) and other neurological conditions in 7.8% (n = 31) among those with two or more anomalies.

**Figure 3:**
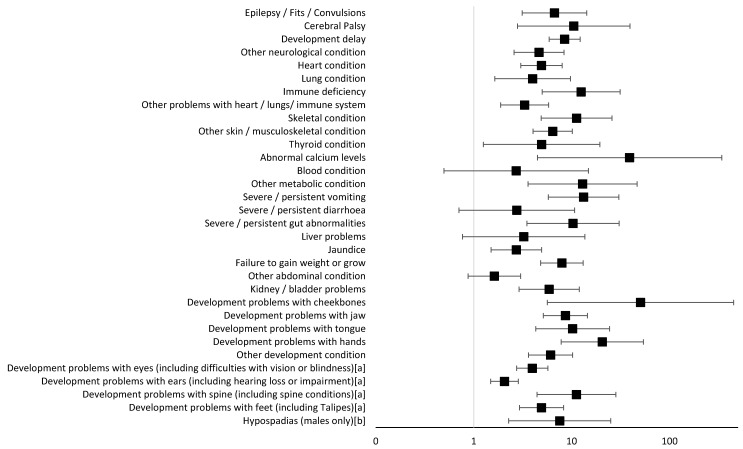
Odds ratios and 95% confidence intervals of the likelihood of being diagnosed with a syndrome (excluding isolated Pierre Robin sequence) when individual anomalies are present versus when they are absent (models adjusted for cleft subtype and biological sex). ^a^ Combined categories; ^b^ males only, n = 983; the solid black line represents the null value.

The diagnosis of a syndrome, excluding Pierre Robin sequence, was made in more than 50% of children when the following anomalies were present: immune deficiency; skeletal condition; severe/persistent vomiting or gut abnormalities; development problems with the jaw, tongue, hands or spine (including spine conditions); and hypospadias (males only). For 27 out of the 32 anomalies tested, strong evidence was found to suggest increased odds of having a syndrome if the anomaly was present compared to if the anomaly was absent (*p*-values ranged between 1.4 × 10^−30^ and 0.002). Odds ratios and 95% confidence intervals are presented in Table 4 and Figure 3. The Bonferroni correction *p*-value cutoff to account for multiple testing was calculated to be 0.0016. The odds of having a syndrome (excluding isolated Pierre Robin sequence) were more than twentyfold if the child identified as having development problems with their hands compared to if the child does not have this anomaly (OR 20.53; 95% CIs 7.82, 53.90; *p* = 8.5 × 10^−10^). The odds of having a syndrome (excluding isolated Pierre Robin sequence) were more than eightfold if the child identified as having developmental delay compared to if the child does not have a developmental delay (OR 8.47; 95% CIs 5.89, 12.20; *p* = 1.4 × 10^−30^). Other notable anomalies, identified in five or more children, suggesting increased odds of over tenfold for being diagnosed with a syndrome (excluding isolated Pierre Robin sequence) comprised the identification of immune deficiency, skeletal conditions, severe or persistent vomiting, severe or persistent gut abnormalities, development problems with the tongue and development problems with the spine. Increased odds of over tenfold for being diagnosed with a syndrome (excluding isolated Pierre Robin sequence) were also seen where a child was diagnosed with cerebral palsy, abnormal calcium levels, other metabolic conditions and development problems with the cheekbones. There is considerable uncertainty over the increased odds of having a syndrome for the following anomalies because the number of children within our sample who were diagnosed with cerebral palsy, abnormal calcium levels, other metabolic conditions and development problems with the cheekbones were small; however, it is possible that these results could still show a clinical significance.

Anomalies that were present in children who had been diagnosed with Stickler syndrome, Van der Woude, 22q11 deletion, craniosynostosis or CHARGE syndrome were described. Developmental delay was reported in all syndromes except Van der Woude. Developmental delay appeared to be most prevalent among children diagnosed with CHARGE syndrome, reported in 83.3% (n = 5) of diagnosed participants (Table S3).

**Figure 4:**
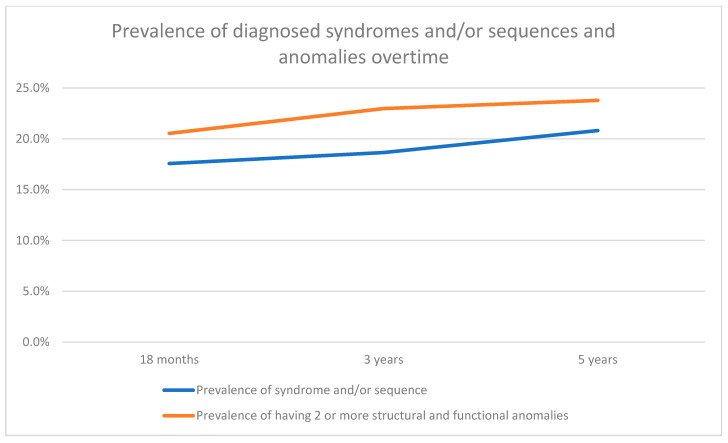
Prevalence of parent-reported diagnosed syndromes and/or sequences and structural and functional anomalies between ages 18 months and 5 years. Note: Due to the possibility of late diagnosis, descriptive analysis on the prevalence of syndromes and structural and functional anomalies were performed at ages 18 months, 3 years and 5 years for a sub-sample of children where data had been received for all three timepoints.

When exploring the trajectory of the diagnosis of a syndrome and/or sequence and the presence of two or more structural and functional anomalies, we saw an increased prevalence with age. In a sub-sample of 370 children, where longitudinal data were available, the prevalence of having a syndrome increased from 17.6% (n = 65) at 18 months to 20.8% (n = 77) at 5 years of age, suggesting 3.2% of syndromes, within our sub-sample, were diagnosed late. Likewise, the presence of two or more anomalies increased from 20.5% (n = 76) at 18 months to 23.8% (n = 88) at 5 years of age (Table S4/Figure 4).

## 4. Discussion

### 4.1. Principal Findings

Data from the Cleft Collective were used to compare syndrome diagnosis between OFC subtypes, biological sex and the presence of two or more structural and functional anomalies. Among our study, the distribution of OFC subtypes and biological sex was similar to that seen in the UK cleft population born between 2020 and 2022 [29], and although we noted some small differences, this is likely due to our sample being born between 2008 and 2022 and due to small levels of sampling error.

Among participants who had two structural and functional anomalies, a diagnosed syndrome and/or sequence was reported in 27.0%, and when excluding isolated Pierre Robin sequence, 16.6% had a diagnosed syndrome. As the number of anomalies present increased so did the proportion of those with a diagnosed syndrome. Among participants who had reported five or more co-occurring anomalies, the proportion of those with a diagnosed syndrome or sequence was 81.5%. The largest difference in the prevalence of anomalies between syndromic and non-syndromic cases was seen in development problems with the jaw (27.3% versus 2.3%, respectively) and developmental delay (30.2% versus 7.2%, respectively). Developmental delay often occurred alongside an additional anomaly (38.3% of cases had developmental delay plus an additional anomaly) and was seen in children with Stickler syndrome, 22q11 deletion, craniosynostosis and CHARGE syndrome.

When exploring the likelihood of having a diagnosed syndrome by anomaly presentation, we excluded those children with isolated Pierre Robin sequence because children with Pierre Robin sequence, by definition, have additional anomalies; also, Pierre Robin sequence is usually diagnosed soon after birth, and those children are likely to be prioritised for genetic testing and hence are more likely to have a syndrome diagnosed if present. We investigated the likelihood of having a syndrome based on the presence of structural and functional anomalies, and for the anomalies we explored, we found that where present the likelihood of having a syndrome was between 19% and 75%. In our cohort, children who had either immune deficiency; skeletal conditions; severe/persistent vomiting; severe/persistent gut abnormalities; failure to thrive; kidney/bladder problems; and developmental problems of the jaw, tongue and hands were more likely to have a syndrome than not. We suspect this would also be true where anomalies with low cell counts were present, but we were unable to report the prevalence of a syndrome in these groups due to disclosure control. Within our logistic regression analysis, there was little to weak statistical evidence to suggest the presence of blood conditions, severe or persistent diarrhoea, liver problems and other abdominal conditions were associated with increased odds of having a syndrome compared to the absence of those conditions. This uncertainty is likely to be due to the low prevalence of each of these anomalies.

### 4.2. Consistency with Other Evidence

Previous studies have reported two or more anomalies occurring within non-syndromic OFC populations at a prevalence of between 6.9% and 21.1% [5,16,30,31]. Our study found the prevalence of two of more anomalies in non-syndromic OFCs was 19.9%, similar to that reported by Sárközi et al. (17.0%) and Pereira et al. (20.8%) [5,31]. Our overall prevalence of two or more anomalies in all OFC cases was 27.8%, which was greater than that reported by Fitzsimons et al. 2022 who found a prevalence of 22.6% [23], and this difference is likely due to our study incorporating a wider range of anomalies, not just congenital anomalies.

Development problems with the ears and eyes were reported in 28.2% and 11.9% of the overall sample, respectively. Comparing the prevalence of development problems with the ears and eyes in the literature is difficult as many published studies have looked broadly at congenital malformations; where development problems with the ears and eyes were included, they were often combined with the face and neck [23]. Developmental delay was reported in 11.9% of our overall sample and within 7.2% of our non-syndromic sample. As above, not all studies in the literature report on developmental delay. An exception is Milerad 1997 who found 18.9% of their non-syndromic sample had developmental delay [30]. It is possible that the difference in the prevalence of developmental delay in non-syndromic OFCs between our study and Milerad 1997 is due to an under-reporting of syndromic OFCs in the latter study. When exploring the presence of multiple anomalies within our study, 38.3% of those with two or more anomalies had reported having developmental delay. Milerad 1997 reported that where multiple anomalies occurred, they often occurred alongside developmental delay [30]. No quantitative data are provided within Milerad 1997 to support this statement.

The anomalies present in participants with syndromes and/or sequences in our sample were reported alongside the anomalies outlined in the published literature [6,7,8,32,33,34,35,36]. Many of the anomalies detailed in the existing evidence were present in our sample. For children diagnosed with Stickler syndrome, 79.0% of our sample reported having difficulties with vision and 42.1% reported hearing loss. A range of skeletal anomalies were also reported in this sample, and these anomalies reflect the anomalies reported in other samples of children born with Stickler syndrome [34]. Additional anomalies seen within our sample of children born with Stickler syndrome included developmental delay and heart conditions; however, numbers were too small to report. Within our sample, anomalies reported in children born with Van der Woude, 22q11 deletion, craniosynostosis and CHARGE syndrome were similar to those reported within the literature [6,7,8,32,33,35,36].

### 4.3. Strengths of This Study

Our sample comprised data from across the UK, and to the authors’ knowledge this is the first study exploring anomalies associated with OFCs which has incorporated data from all four nations.

Data were available on a wide range of anomalies. Many previous studies have focused upon congenital anomalies associated with OFCs, whereas this study included additional anomalies which may not be apparent until later in childhood yet still may be associated with OFCs. Congenital anomalies such as heart conditions, cerebral palsy, talipes and hypospadias were also included within the anomalies analysed.

Analyses were stratified by and/or adjusted for OFC subtype, syndromic status and biological sex. All four OFC subtypes were included. This stratification enables us to identify whether the prevalence of anomalies differs by subtype given that previous evidence suggests differing aetiologies [2,12,37,38,39]. These results will help inform genetic analysis within the Cleft Collective and identify characteristics that will help determine when genetic screening should be prioritised.

### 4.4. Limitations of the Data

Some anomalies listed within the parental follow-up questionnaires might be considered vague and/or ambiguous. To reduce the possibility of counting the same anomaly twice, some were combined. It is possible that some parents may have selected development problems with their child’s ears because their child had been diagnosed with OME. However, the prevalence of OME was almost twice as common as the prevalence of development problems of the ears, and because OME is a common occurrence among children, this was excluded from our analysis. The anomalies listed within the questionnaires are extensive, but they are not exhaustive, and some anomalies may not have been captured.

There is a possibility that certain anomalies explored within our analyses may trigger genetic testing more so than others. As a result, it is possible that our results may be impacted by ascertainment bias as children presenting with these anomalies are more likely to have received a diagnosis if they have undergone genetic testing. Additionally, where a child has undergone genetic testing due to family history and/or more routine testing at their cleft centre, a syndrome may have been diagnosed yet anomalies may not have been identified at the time data were collected.

The diagnosis of a syndrome and/or structural and functional anomaly were parent-reported. Where a diagnosis of a syndrome or presence of an anomaly has been made, it is thought that the parental report is generally accurate [40,41]. The presence of molecular confirmation of a monogenic syndrome diagnosis, for example, was not confirmed. Furthermore, some syndromes are not diagnosed until the child is older or not diagnosed at all meaning that the presence of a syndrome may be unknown.

Data were missing for 54% of the overall Cleft Collective cohort due to the questionnaire response rate. We were unable to perform multiple imputation since predictors of the structural and functional anomalies included within our analyses have not been reported in the current literature. It is possible that the missing data could have resulted in selection bias; however, when comparing the characteristics of our sample to the CRANE database, there appeared to be little discrepancy in OFC subtype and sex distribution between the samples suggesting that our sample is representative of the population of interest.

## 5. Interpretation—Clinical and Research Implications

The findings of this study have both clinical and research implications.

Defining the role of Clinical Genetics within the multidisciplinary cleft team is challenging. On the one hand, the prompt identification and diagnosis of individuals with underlying syndromes is clearly desirable, since molecular diagnosis can support the implementation of tailored medical care, facilitate access to appropriate peer support and enable accurate genetic counselling of parents with regard to recurrence risks in future pregnancy. Conversely, a comprehensive genetic investigation of those whose OFC has a likely multifactorial basis may be costly, with low predicted yield, as well as potentially raising anxiety among families including the possibility of uncertain or incidental findings from genomic tests.

Knowledge of which anomalies most increase the odds of having a diagnosed syndrome can inform clinical practice to ensure that those individuals with higher predicted yield from genetic investigation are prioritised to be offered genetic counselling and testing. From our data, the presence of two or more additional anomalies and/or immune deficiency; skeletal conditions; severe/persistent vomiting or gut abnormalities; development problems with the jaw, tongue, hands or spine (including spine conditions); and hypospadias (males only) could be considered useful triggers to recommend genetic assessment.

It should be noted that, even with extensive genetic investigations such as trio exome sequencing, the diagnostic yield among children with a suspected monogenic developmental disorders is only around 41% [42]. The reasons for negative results may include the presence of cryptic mutations affecting known genes, mutations within novel genes or more complex inheritance patterns. It is therefore highly probable that cleft cohorts include individuals who have monogenic syndromes but who lack a specific molecular diagnosis. We recommend that future research should include a “suspected syndrome” category for those children with an OFC who have not been diagnosed with a syndrome and/or sequence but who have multiple anomalies and/or anomalies which are associated with a high prevalence among syndromic cases. The inclusion of a “suspected syndrome” will enable us to reduce “noise” within future analyses and will allow for targeted genetic analyses which in turn will further our understanding of the complex aetiology of OFCs.

## 6. Conclusions

It is important to identify the prevalence of commonly occurring anomalies and the anomalies which may predict the presence of a syndrome to help inform clinical practice. Common anomalies included development problems with the ears and eyes and developmental delay. Anomalies which increased the odds of having a diagnosed syndrome included developmental delay and developmental problems affecting the hands. Clinicians caring for children with an OFC should be aware of the common associated anomalies to inform clinical assessment and management, as well as to identify those individuals who are most likely to benefit from genetic counselling and testing. Children with an OFC and two or more anomalies present or anomalies which present with a high prevalence of syndromic cases should be prioritised and offered assessment by Clinical Genetics services.

## Figures and Tables

**Table 1:** Sample characteristics: distribution of cleft subtype and biological sex by syndromic status (syndrome and/or sequence present).

	Characteristics Within the Cleft Collective					
	Overall n = 1701		No Syndrome n = 1353 (79.5%)		Syndrome and/or PRS n = 348 (20.5%)	
**Cleft Type**						
Cleft lip	423		395 (93.4%)		28 (6.6%)	
Cleft palate	653		401 (61.4%)		252 (38.6%)	
Unilateral cleft lip and palate	446		410 (91.9%)		36 (8.1%)	
Bilateral cleft lip and palate	179		147 (82.1%)		32 (17.9%)	
**Biological sex**						
Male	983		821 (83.5%)		162 (16.5%)	
Female	718		532 (74.1%)		186 (25.9%)	
**Cleft Type**	**Male**	**Female**	**Male**	**Female**	**Male**	**Female**
Cleft lip	260	163	242 (93.1%)	153 (93.9%)	18 (6.9%)	10 (6.1%)
Cleft palate	282	371	180 (63.8%)	221 (59.6%)	102 (36.2%)	150 (40.4%)
Unilateral cleft lip and palate	310	136	290 (93.6%)	120 (88.2%)	20 (6.5%)	16 (11.6%)
Bilateral cleft lip and palate	131	48	109 (83.2%)	38 (79.2%)	22 (16.8%)	10 (20.8%)

Pierre Robin sequence (PRS); percentages for cleft type and biological sex by syndromic status show the proportion of children without a diagnosed syndrome and those with a diagnosed syndrome by cleft subtype and biological sex (row percentages); percentages for cleft type by biological sex and syndromic status show the proportion of children with and without a syndrome by biological sex and cleft type.

**Table 2:** Syndromic diagnosis by the number of co-occurring structural and functional anomalies.

Number of Structural and Functional Anomalies Identified	Number of Children Overall [n = 1701]	Number of Children with a Syndrome and/or Sequence (%) [n = 348]	Number of Children Excluding Isolated PRS [n = 1546]	Number of Children with a Syndrome Excluding Isolated PRS (%) [n = 193]
0	762	62 (8.1%)	728	28 (3.8%)
1	466	82 (17.6%)	418	34 (8.1%)
2	200	54 (27.0%)	175	29 (16.6%)
3	115	42 (36.5%)	90	17 (18.9%)
4	66	33 (50.0%)	56	23 (41.1%)
5 or more	92	75 (81.5%)	79	62 (78.5%)

Pierre Robin sequence (PRS); row percentages are shown.

**Table 3:** Prevalence of structural and functional anomalies by syndromic status.

	Syndromic Status								
	No Syndrome or Sequence (n = 1353)			Syndrome or Sequence (n = 348)			Syndrome Excluding Isolated PRS (n = 193)		
**Number of Structural and Functional** **Anomalies Present**	**n**	**Prevalence (95% CIs) ^a^**		**n**	**Prevalence (95% CIs) ^a^**		**n**	**Prevalence (95% CIs) ^a^**	
0	700	0.52	(0.49, 0.54)	62	0.18	(0.14, 0.22)	28	0.15	(0.10. 0.20)
1	384	0.28	(0.26, 0.31)	82	0.24	(0.19, 0.28)	34	0.18	(0.12, 0.23)
2 or more	269	0.20	(0.18, 0.22)	204	0.59	(0.53, 0.64)	131	0.68	(0.61, 0.75)
**Individual Structural and Functional** **Anomalies**	**n**	**Prevalence (95% CIs) ^a^**		**n**	**Prevalence (95% CIs) ^a^**			**Prevalence (95% CIs) ^a^**	
Epilepsy/fits/convulsions	17	0.01	(0.01, 0.02)	***			14	0.07	(0.04, 0.11)
Cerebral palsy	<5			<5			<5		
Developmental delay	98	0.07	(0.06, 0.09)	105	0.30	(0.25, 0.35)	80	0.41	(0.34, 0.48)
Other neurological condition	34	0.03	(0.02, 0.03)	29	0.08	(0.05, 0.11)	21	0.11	(0.06, 0.15)
Heart condition	48	0.04	(0.03, 0.05)	47	0.14	(0.10, 0.17)	33	0.17	(0.12, 0.22)
Lung condition	13	0.01	(0.004, 0.02)	***			9	0.05	(0.02, 0.08)
Immune deficiency	8	0.01	(0.002, 0.01)	***			14	0.07	(0.04, 0.11)
Other problems with heart/lungs/immune system	47	0.04	(0.03, 0.05)	27	0.08	(0.05, 0.11)	20	0.10	(0.06, 0.15)
Skeletal condition	9	0.01	(0.002, 0.01)	***			18	0.09	(0.05, 0.13)
Other skin/musculoskeletal condition	52	0.04	(0.03, 0.05)	***			40	0.21	(0.15, 0.26)
Thyroid condition	<5			<5			<5		
Abnormal calcium levels	<5			<5			<5		
Blood condition	<5			<5			<5		
Other metabolic condition	<5			<5			<5		
Severe/persistent vomiting	9	0.01	(0.002, 0.01)	25	0.07	(0.05, 0.10)	19	0.10	(0.06, 0.14)
Severe/persistent diarrhoea	<5			<5			<5		
Severe/persistent gut abnormalities	6	0.004	(0.001, 0.01)	***			9	0.05	(0.02, 0.08)
Liver problems	<5			<5			<5		
Jaundice	47	0.04	(0.025, 0.05)	25	0.07	(0.05, 0.10)	17	0.09	(0.05, 0.13)
Failure to gain weight or grow	38	0.03	(0.02, 0.04)	51	0.15	(0.11, 0.18)	37	0.19	(0.14, 0.25)
Other abdominal condition	59	0.04	(0.03, 0.05)	21	0.06	(0.04, 0.09)	14	0.07	(0.04, 0.11)
Kidney/bladder problems	19	0.01	(0.01, 0.02)	22	0.06	(0.04, 0.09)	16	0.08	(0.04, 0.12)
Development problems with cheekbones	<5			<5			<5		
Development problems with jaw	31	0.02	(0.02, 0.03)	95	0.27	(0.23, 0.32)	38	0.20	(0.14, 0.25)
Development problems with tongue	9	0.01	(0.002, 0.01)	28	0.08	(0.05, 0.11)	15	0.08	(0.04, 0.12)
Development problems with hands	6	0.004	(0.001, 0.01)	***			18	0.09	(0.05, 0.13)
Other development condition	38	0.03	(0.02, 0.04)	40	0.12	(0.08, 0.15)	31	0.16	(0.11, 0.21)
*Combined categories*									
Development problems with eyes (including difficulties with vision or blindness)	124	0.09	(0.08, 0.11)	79	0.23	(0.18, 0.27)	59	0.31	(0.24, 0.37)
Development problems with ears (including hearing loss or impairment)	335	0.25	(0.23, 0.27)	145	0.42	(0.37, 0.47)	83	0.43	(0.36, 0.50)
Development problems with spine (including spine conditions)	8	0.01	(0.002, 0.01)	***			13	0.07	(0.24, 0.37)
Development problems with feet (including talipes)	40	0.03	(0.02, 0.04)	37	0.11	(0.07, 0.14)	29	0.15	(0.10, 0.20)
*Biological sex-specific conditions*	***n = 821***			***n = 162***			***n = 107***		
Hypospadias (males only n = 983)	6	0.01	(0.001, 0.01)	***			6	0.06	(0.01, 0.10)

^a^ 95% confidence intervals (95% CIs) refer to the estimate of the prevalence within the population of interest; <5 at least one cell is disclosive by count or deduction; *** cell is disclosive by deduction.

**Table 4:** Logistic regression exploring the odds of having a syndrome (excluding isolated Pierre Robin Sequence) for each structural and functional anomaly present versus absent anomaly, with models adjusted for cleft subtype and biological sex (overall n = 1546).

Individual Structural and Functional Anomalies (n)	Prevalence of Children Diagnosed with a Syndrome (Excluding Isolated PRS) If Specified Anomaly Has Been Identified	Diagnosed Syndrome (Excluding Isolated PRS)			Diagnosed and Suspected Syndrome ^d^ Combined (Excluding Isolated PRS)		
		Odds Ratio (OR) ^c^	*p* (ϐ = 0.001563)	95% CIs	Odds Ratio (OR) ^c^	*p* (ϐ = 0.001563)	95% CIs
Epilepsy/fits/convulsions (n = 31)	0.45	6.67	9.20 × 10^−7^	3.13, 14.23	9.92	2.90 × 10^−7^	4.13, 23.83
Cerebral palsy (n = 10)	¥	10.49	5.00 × 10^−4^	2.80, 39.39	*		
Developmental delay (n = 178)	0.45	8.47	1.40 × 10^−30^	5.89, 12.20	32.48	7.30 × 10^−39^	19.25, 54.80
Other neurological condition (n = 55)	0.38	4.64	3.10 × 10^−7^	2.58, 8.35	18.85	2.00 × 10^−12^	8.32, 42.74
Heart condition (n = 81)	0.41	4.92	1.40 × 10^−10^	3.02, 7.99	14.98	6.60 × 10^−17^	7.94, 28.26
Lung condition (n = 22)	0.41	3.99	2.30 × 10^−3^	1.64, 9.70	13.82	3.40 × 10^−5^	3.99, 47.87
Immune deficiency (n = 22)	0.64	12.49	6.60 × 10^−8^	4.99, 31.21	*		
Other problems with heart/lungs/immune system (n = 67)	0.30	3.31	3.30 × 10^−5^	1.88, 5.82	13.52	5.70 × 10^−15^	7.03, 25.98
Skeletal condition (n = 27)	0.67	11.23	1.20 × 10^−8^	4.89, 25.80	50.36	1.30 × 10^−4^	6.78, 374.18
Other skin/musculoskeletal condition (n = 92)	0.43	6.40	2.90 × 10^−15^	4.04, 10.14	17.25	6.00 × 10^−20^	9.37, 31.76
Thyroid condition (n = 9)	¥	4.93	0.022	1.25, 19.37	4.55	0.039	1.08, 19.11
Abnormal calcium levels (n = 7)	¥	39.02	9.10 × 10^−4^	4.47, 340.17	*		
Blood condition (n = 7)	¥	2.71	0.249	0.50, 14.83	15.92	0.012	1.83, 138.40
Other metabolic condition (n = 11)	¥	12.92	9.20 × 10^−5^	3.58, 46.59	*		
Severe/persistent vomiting (n = 28)	0.68	13.21	1.10 × 10^−9^	5.76, 30.28	17.55	3.90 × 10^−6^	5.20, 59.21
Severe/persistent diarrhoea (n = 12)	¥	2.75	0.145	0.71, 10.72	7.99	2.40 × 10^−3^	2.09, 30.57
Severe/persistent gut abnormalities (n = 15)	0.60	10.31	2.40 × 10^−5^	3.49, 30.46	32.98	8.30 × 10^−4^	4.25, 256.07
Liver problems (n = 9)	¥	3.24	0.109	0.77, 13.63	7.63	0.013	1.54, 37.72
Jaundice (n = 64)	0.27	2.73	9.40 × 10^−4^	1.51, 4.94	5.05	5.50 × 10^−9^	2.93, 8.70
Failure to gain weight or grow (n = 75)	0.49	7.94	4.80 × 10^−16^	4.82, 13.10	17.07	5.60 × 10^−16^	8.59, 33.92
Other abdominal condition (n = 73)	0.19	1.62	0.125	0.87, 3.01	11.37	4.30 × 10^−15^	6.20, 20.88
Kidney/bladder problems (n = 35)	0.46	5.89	8.90 × 10^−7^	2.90, 11.94	5.97	4.30 × 10^−6^	2.79, 12.81
Development problems with cheekbones (n = 6)	¥	50.39	4.50 × 10^−4^	5.64, 450.27	*		
Development problems with jaw (n = 69)	0.55	8.62	3.90 × 10^−16^	5.13, 14.49	14.00	6.90 × 10^−14^	7.02, 27.93
Development problems with tongue (n = 24)	0.63	10.22	1.50 × 10^−7^	4.29, 24.34	48.59	1.60 × 10^−4^	6.48, 364.56
Development problems with hands (n = 24)	0.75	20.53	8.50 × 10^−10^	7.82, 53.90	*		
Other development condition (n = 69)	0.45	6.08	8.60 × 10^−12^	3.62, 10.22	15.59	8.40 × 10^−15^	7.79, 31.19
Development problems with eyes (including difficulties with vision or blindness) ^a^ (n = 183)	0.32	3.96	2.50 × 10^−13^	2.74, 5.72	8.40	3.50 × 10^−31^	5.87, 12.03
Development problems with ears (including hearing loss or impairment) ^a^ (n = 418)	0.20	2.06	1.40 × 10^−5^	1.49, 2.86	6.92	1.00 × 10^−47^	5.33, 8.98
Development problems with spine (including spine conditions) ^a^ (n = 21)	0.62	11.18	3.40 × 10^−7^	4.42, 28.27	*		
Development problems with feet (including talipes) ^a^ (n = 69)	0.42	4.92	1.80 × 10^−9^	2.93, 8.26	13.47	2.50 × 10^−14^	6.90, 26.30
Hypospadias (males only) ^b^ (n = 12)	0.50	7.54	9.50 × 10^−4^	2.27, 25.01	7.21	4.40 × 10^−3^	1.85, 28.08

ϐ—Bonferroni correction; 95% CIs—95% confidence intervals; ^a^ combined categories; ^b^ males only, n = 983; ^c^ odds of having a syndrome if anomaly is present versus if absent; ^d^ syndrome suspected where child has two or more structural and functional anomalies; ¥ prevalence cannot be reported due to cell counts of less than 5; * model omitted due to collinearity; Pierre Robin sequence (PRS).

## Data Availability

Data from the Cleft Collective Cohort Studies were used within the analyses presented. The Cleft Collective is a resource available to clinicians and researchers globally to be able to answer cleft-related research questions. Details on how to access the data can be found at https://www.bristol.ac.uk/cleft-collective/professionals/access/, accessed on 15 October 2024.

## Supplementary Materials

The following are available online at https://www.mdpi.com/article/10.3390/jcm13226924/s1, Supplementary File S1—Syndrome status and structural and functional anomalies questions within Cleft Collective follow-up questionnaires, Table S1—Characteristics of the Cleft Collective sample and children born with a cleft between 2020 and 2022 who were registered in CRANE, Figure S1—Distribution of cleft subtype by syndromic status, Figure S2—Odds of having a syndrome and/or sequence by orofacial cleft subtype and biological sex, Table S2a—Prevalence of structural and functional anomalies—overall sample n = 1701, Table S2b—Prevalence of structural and functional anomalies by cleft subtype, Table S2c—Prevalence of structural and functional anomalies by syndromic status and biological sex, Table S3—Common syndromes associated with cleft lip and palate and the anomalies seen in participants of the Cleft Collective, Table S4—Prevalence of parent-reported diagnosed syndromes and structural and functional anomalies between ages 18 months and 5 years.

## Abbreviations

OFCs	Orofacial clefts
UK	United Kingdom of Great Britain and Northern Ireland
CRANE	The Cleft Registry and Audit NEtwork (CRANE)
