# Supplementary material for: Predicting Syndromic Status Based on Longitudinal Data from Parental Reports of the Presence of Additional Structural and Functional Anomalies in Children Born with an Orofacial Cleft
Source: J Clin Med. 2024 Nov 17;13(22):6924. doi: 10.3390/jcm13226924 (PMC11594774; doi:10.3390/jcm13226924)
Supplement: Supplementary file 1 [file jcm-13-06924-s001.zip › jcm-3289865-supplementary.pdf]

Predicting syndromic status based on the presence of additional structural and functional anomalies in children born with an orofacial cleft - Supplementary Material

File S1 – Syndrome status and structural and functional anomalies questions within Cleft Collective follow-up questionnaires

Table S1 – Characteristics of the Cleft Collective sample and children born with a cleft between 2020-2022 who were registered in CRANE

Figure S1 – Distribution of cleft subtype by syndromic status

Figure S2 – Odds of having a syndrome and/or sequence by orofacial cleft subtype and biological sex

Table S2a – Prevalence of structural and functional anomalies – overall sample n=1701

Table S2b – Prevalence of structural and functional anomalies by cleft subtype

Table S2c – Prevalence of structural and functional anomalies by syndromic status and biological sex

Table S3– Common syndromes associated with cleft lip and palate and the anomalies seen in participants of the Cleft Collective

Table S4 – Prevalence of parent reported diagnosed syndromes and structural and functional anomalies between ages 18 months and 5 years

File S1 – Syndrome status and structural and functional anomalies questions within Cleft Collective follow-up questionnaires

Syndromes and sequences

Has your child been diagnosed with any of the following syndromes / genetic conditions?

(Cross all that apply)

a) Pierre Robin sequence (PRS)
b) Van der Woude syndrome
c) Treacher Collins syndrome
d) Hemifacial Microsomy / Goldenhar syndrome
e) Stickler syndrome
f) 22q 11.2 deletion syndrome (also known as Velocardiofacial syndrome, Shprintzen syndrome, DiGeorge syndrome)
g) Craniosynostosis (including Crouzon syndrome, Apert syndrome, Pfeiffer syndrome, Saethre-Chotzen syndrome)
h) Cornelia de Lange syndrome
i) Other syndrome / genetic condition (specify)
j) We are currently undergoing genetic testing at the hospital
k) None of the above

Structural and functional anomalies

Has your child had / does your child have any of the following conditions or problems?

(Cross all that apply)

a) Neurological / sensory conditions
   I. None
   II. Epilepsy / fits / convulsions
   III. Cerebral Palsy
   IV. Developmental delay
   V. Hearing loss or impairment
   VI. Glue ear

VII. Difficulties with vision / blindness
VIII. Other neurological conditions

b) Heart / Lungs / Immune system

I. None
II. Heart conditions
III. Lung conditions
IV. Asthma / difficulties breathing
V. Allergies
VI. Immune deficiency
VII. Other problems with heart / lungs / immune system

c) Skin / musculoskeletal conditions

I. None
II. Skin condition
III. Skeletal condition
IV. Talipes (club foot)
V. Spine condition
VI. Other skin / musculoskeletal condition

d) Metabolic conditions

I. None
II. Thyroid condition
III. Abnormal calcium levels
IV. Blood condition
V. Other metabolic conditions

e) Abdominal conditions

I. None
II. Severe / persistent vomiting
III. Severe / persistent diarrhoea
IV. Severe / persistent gut abnormalities
V. Liver problems
VI. Jaundice
VII. Failure to gain weight or grow
VIII. Other abdominal conditions

f) Kidney and bladder problems

I. None

II. Kidney / bladder problems
III. Hypospadias (males only)

Does your child have problems with the development of any of the following? (Cross all that apply)

a) Eyes
b) Ears
c) Cheekbones
d) Jaw
e) Tongue
f) Hands
g) Feet
h) Spine
i) Other developmental condition
j) None of the above

Full copies of follow-up questionnaires provided to participants within the Cleft Collective can be found here:

https://www.bristol.ac.uk/dental/cleft-collective/professionals/information/questionnaires/

[Accessed on 15 October 2024]

Table S1 – Characteristics of the Cleft Collective sample and children born with a cleft between 2020-2022 who were registered in CRANE

	**Characteristics within the Cleft Collective**	**Characteristics reported within the CRANE 2023 Annual Report***
**Cleft Type**	**(n=1701)**	**(n=2403)**
Cleft lip	423 (24.9%)	624 (26.0%)
Cleft palate	653 (38.4%)	1032 (42.9%)
Unilateral cleft lip and palate	446 (26.2%)	516 (21.5%)
Bilateral cleft lip and palate	179 (10.5%)	231 (9.6%)
**Biological sex**	**(n=1701)**	**(n=2625)**
Male	983 (57.8%)	1497 (57.0%)
Female	718 (42.2%)	1128 (43.0%)

Cleft Registry & Audit NEtwork (CRANE); Characteristics of CRANE sample (children born within England, Wales and Northern Ireland with a cleft between 2020-2023 and registered within the CRANE database by 4th July 2023) reported within the CRANE 2023 Annual Report, provided for comparative reasons to show similarities of distribution of cleft subtype and biological sex, suggesting limited selection bias within the Cleft Collective sample.

Figure S1 – Distribution of cleft subtype by syndromic status

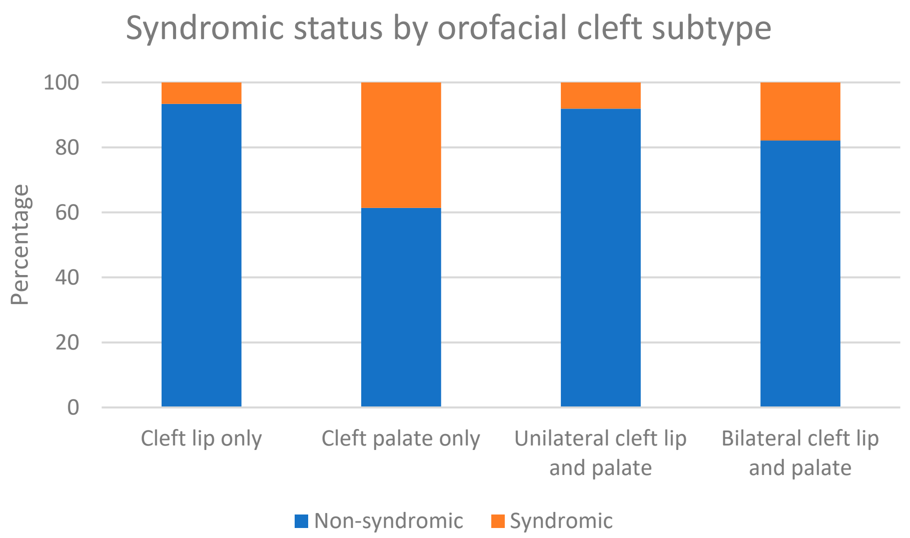

Where syndromic status is based on the presence of having a syndrome and/or a sequence

Figure S2 – Odds of having a syndrome and/or sequence by orofacial cleft subtype and biological sex

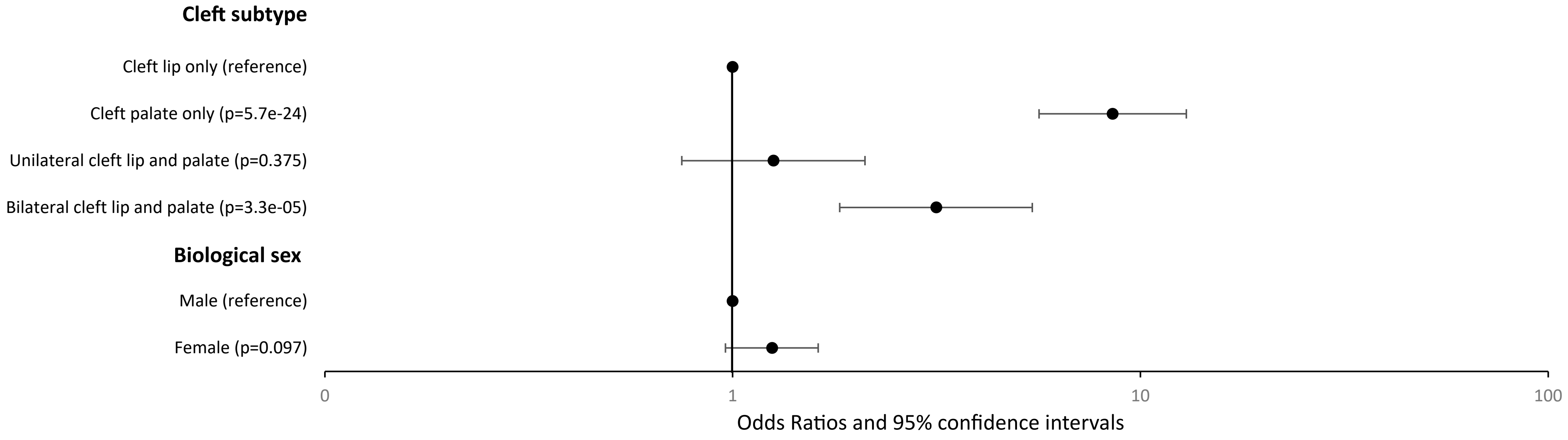

*The solid vertical black line represents the null value; Odds of having a syndrome by orofacial cleft subtype were adjusted for biological sex; Odds of having a syndrome by biological sex were adjusted for orofacial cleft subtype

Table S2a – Prevalence of structural and functional anomalies – overall sample n=1701

	Overall sample (n=1701)		
**Number of structural and functional anomalies present**	**n**	**Prevalence (95% CIs)[a]**	
0	762	0.45	(0.42. 0.47)
1	466	0.28	(0.25. 0.30)
2 or more	473	0.28	(0.26. 0.30)
**Individual structural and functional anomalies**	**n**	**Prevalence (95% CIs)[a]**	
Epilepsy / Fits / Convulsions	35	0.02	(0.01, 0.03)
Cerebral Palsy	10	0.01	(0.002, 0.01)
Developmental delay	203	0.12	(0.10, 0.14)
Other neurological condition	63	0.04	(0.03, 0.05)
Heart condition	95	0.06	(0.05, 0.07)
Lung condition	24	0.01	(0.01, 0.02)
Immune deficiency	25	0.02	(0.01, 0.02)
Other problems with heart / lungs/ immune system	74	0.04	(0.03, 0.05)
Skeletal condition	29	0.02	(0.01, 0.02)
Other skin / musculoskeletal condition	94	0.06	(0.04, 0.07)
Thyroid condition	9	0.01	(0.002, 0.01)
Abnormal calcium levels	7	0.004	(0.001, 0.01)
Blood condition	8	0.01	(0.001, 0.01)
Other metabolic condition	12	0.01	(0.003, 0.01)
Severe / persistent vomiting	34	0.02	(0.01, 0.03)
Severe / persistent diarrhoea	13	0.01	(0.004, 0.01)
Severe / persistent gut abnormalities	18	0.01	(0.01, 0.02)
Liver problems	10	0.01	(0.002, 0.01)
Jaundice	72	0.04	(0.03, 0.05)
Failure to gain weight or grow	89	0.05	(0.04, 0.06)
Other abdominal condition	80	0.05	(0.04, 0.06)
Kidney / bladder problems	41	0.02	(0.02, 0.03)
Development problems with cheekbones	6	0.004	(0.001, 0.01)
Development problems with jaw	126	0.07	(0.06, 0.09)
Development problems with tongue	37	0.02	(0.02, 0.03)
Development problems with hands	27	0.02	(0.01, 0.02)
Other development condition	78	0.05	(0.04, 0.06)
Combined categories			
Development problems with eyes (including difficulties with vision or blindness)	203	0.12	(0.10, 0.14)
Development problems with ears (including hearing loss or impairment)	480	0.28	(0.26, 0.30)
Development problems with spine (including spine conditions)	24	0.01	(0.01, 0.02)
Development problems with feet (including Talipes)	77	0.05	(0.04, 0.06)
Biological sex specific conditions			

[a]95% confidence intervals refer to the estimate of the prevalence within the population of interest

Hypospadias (males only n=983)	15	0.02	(0.01, 0.02)

[a]95% confidence intervals refer to the estimate of the prevalence within the population of interest; <5 at least one cell is disclosive by count or deduction

Table S2b – Prevalence of structural and functional anomalies by cleft subtype

	Cleft lip only (n=423)			Cleft palate only (n=653)			Unilateral cleft lip and palate (n=446)			Bilateral cleft lip and palate (n=179)		
**Number of structural and functional anomalies present**	**n**	**Prevalence (95% CIs)[a]**		**n**	**Prevalence (95% CIs)[a]**		**n**	**Prevalence (95% CIs)[a]**		**n**	**Prevalence (95% CIs)[a]**	
0	264	0.62	(0.58, 0.67)	225	0.35	(0.31, 0.38)	200	0.45	(0.40, 0.50)	73	0.41	(0.34, 0.48)
1	105	0.25	(0.21, 0.29)	185	0.28	(0.25, 0.32)	127	0.29	(0.24, 0.33)	49	0.27	(0.21, 0.34)
2 or more	54	0.13	(0.10, 0.16)	243	0.37	(0.34, 0.41)	119	0.27	(0.23, 0.31)	57	0.32	(0.25, 0.39)
**Individual structural and functional anomalies**	**n**	**Prevalence (95% CIs)[a]**		**n**	**Prevalence (95% CIs)[a]**		**n**	**Prevalence (95% CIs)[a]**		**n**	**Prevalence (95% CIs)[a]**	
Epilepsy / Fits / Convulsions	10	0.02	(0.01, 0.04)	12	0.02	(0.01, 0.03)	7	0.02	(0.004, 0.03)	6	0.03	(0.01, 0.06)
Cerebral Palsy	<5			<5			<5			<5		
Developmental delay	27	0.06	(0.04, 0.09)	107	0.16	(0.14, 0.19)	45	0.10	(0.07, 0.13)	24	0.13	(0.08, 0.18)
Other neurological condition	12	0.03	(0.01, 0.04)	29	0.04	(0.03, 0.06)	13	0.03	(0.01, 0.05)	9	0.05	(0.02, 0.08)
Heart condition	13	0.03	(0.01, 0.05)	51	0.08	(0.06, 0.10)	17	0.04	(0.02, 0.06)	14	0.08	(0.04, 0.12)
Lung condition	<5			14	0.02	(0.01, 0.03)	<5			<5		
Immune deficiency	<5			12	0.02	(0.01, 0.03)	<5			<5		
Other problems with heart / lungs/ immune system	16	0.04	(0.02, 0.06)	28	0.04	(0.03, 0.06)	20	0.05	(0.03, 0.06)	10	0.06	(0.02, 0.09)
Skeletal condition	<5			18	0.03	(0.02, 0.04)	<5			7	0.04	(0.01, 0.07)
Other skin / musculoskeletal condition	17	0.04	(0.02, 0.06)	39	0.06	(0.04, 0.08)	25	0.06	(0.04, 0.08)	13	0.07	(0.03, 0.11)
Thyroid condition	<5			<5			<5			<5		
Abnormal calcium levels	<5			<5			<5			<5		
Blood condition	<5			<5			<5			<5		
Other metabolic condition	<5			5	0.01	(0.001, 0.01)	<5			<5		
Severe / persistent vomiting	<5			22	0.03	(0.02, 0.05)	<5			<5		
Severe / persistent diarrhoea	<5			5	0.01	(0.001, 0.01)	5	0.01	(0.001, 0.02)	<5		
Severe / persistent gut abnormalities	<5			11	0.02	(0.01, 0.03)	<5			<5		
Liver problems	<5			5	0.01	(0.001, 0.01)	<5			<5		
Jaundice	15	0.04	(0.02, 0.05)	27	0.04	(0.03, 0.06)	19	0.04	(0.02, 0.06)	11	0.06	(0.03, 0.10)
Failure to gain weight or grow	12	0.03	(0.01, 0.04)	46	0.07	(0.05, 0.09)	20	0.05	(0.03, 0.06)	11	0.06	(0.03, 0.10)
Other abdominal condition	14	0.03	(0.02, 0.05)	34	0.05	(0.04, 0.07)	22	0.05	(0.03, 0.07)	10	0.06	(0.02, 0.09)
Kidney / bladder problems	7	0.02	(0.004, 0.03)	19	0.03	(0.02, 0.04)	8	0.02	(0.01, 0.03)	7	0.04	(0.01, 0.07)
Development problems with cheekbones	<5			<5			<5			<5		

Development problems with jaw	7	0.02	(0.004, 0.03)	101	0.16	(0.13, 0.18)	13	0.03	(0.01, 0.05)	5	0.03	(0.004, 0.05)
Development problems with tongue	<5			29	0.04	(0.03, 0.06)	5	0.01	(0.001, 0.02)	<5		
Development problems with hands	<5			19	0.03	(0.02, 0.04)	<5			<5		
Other development condition	13	0.03	(0.01, 0.05)	38	0.06	(0.04, 0.08)	14	0.03	(0.02, 0.05)	13	0.07	(0.03, 0.11)
Combined categories												
Development problems with eyes (including difficulties with vision or blindness)	34	0.08	(0.05, 0.11)	106	0.16	(0.13, 0.19)	45	0.10	(0.07, 0.13)	18	0.10	(0.06, 0.15)
Development problems with ears (including hearing loss or impairment)	31	0.07	(0.05, 0.10)	225	0.35	(0.31, 0.38)	161	0.36	(0.32, 0.41)	63	0.35	(0.28, 0.42)
Development problems with spine (including spine conditions)	<5			14	0.02	(0.01, 0.03)	7	0.02	(0.004, 0.03)	<5		
Development problems with feet (including Talipes)	12	0.03	(0.01, 0.04)	43	0.07	(0.05, 0.09)	11	0.03	(0.01, 0.04)	11	0.06	(0.03, 0.10)
Biological sex specific conditions	***n=260***			***n=282***			***n=310***			***n=131***		
Hypospadias (males only n=983)	<5			6	0.02	(0.004, 0.04)	<5			<5		

[a]95% confidence intervals refer to the estimate of the prevalence within the population of interest; <5 at least one cell is disclosive by count or deduction

Table S2c – Prevalence of structural and functional anomalies by biological sex

	Biological sex					
	Male (n=983)			Female (n=718)		
**Number of structural and functional anomalies present**	**n**	**Prevalence (95% CIs)[a]**		**n**	**Prevalence (95% CIs)[a]**	
0	435	0.44	(0.41, 0.47)	327	0.46	(0.42, 0.49)
1	270	0.28	(0.25, 0.30)	196	0.27	(0.24, 0.31)
2 or more	278	0.28	(0.26, 0.31)	195	0.27	(0.24, 0.30)
**Individual structural and functional anomalies**	**n**	**Prevalence (95% CIs)[a]**		**n**	**Prevalence (95% CIs)[a]**	
Epilepsy / Fits / Convulsions	20	0.02	(0.01, 0.03)	15	0.02	(0.01, 0.03)
Cerebral Palsy	<5			<5		
Developmental delay	129	0.13	(0.11, 0.15)	74	0.10	(0.08, 0.13)
Other neurological condition	44	0.05	(0.03, 0.06)	19	0.03	(0.02, 0.04)
Heart condition	52	0.05	(0.04, 0.07)	43	0.06	(0.04, 0.08)
Lung condition	15	0.02	(0.01, 0.02)	9	0.01	(0.004, 0.02)
Immune deficiency	14	0.01	(0.01, 0.02)	11	0.02	(0.01, 0.02)
Other problems with heart / lungs/ immune system	41	0.04	(0.03, 0.05)	33	0.05	(0.03, 0.06)
Skeletal condition	15	0.02	(0.01, 0.02)	14	0.02	(0.01, 0.03)
Other skin / musculoskeletal condition	60	0.06	(0.05, 0.08)	34	0.05	(0.03, 0.06)
Thyroid condition	<5			<5		
Abnormal calcium levels	<5			<5		
Blood condition	<5			<5		
Other metabolic condition	6	0.01	(0.001, 0.01)	6	0.01	(0.002, 0.02)
Severe / persistent vomiting	16	0.02	(0.01, 0.02)	18	0.03	(0.01, 0.04)
Severe / persistent diarrhoea	<5			<5		
Severe / persistent gut abnormalities	8	0.01	(0.003, 0.01)	10	0.01	(0.01, 0.02)
Liver problems	<5			<5		
Jaundice	41	0.04	(0.03, 0.05)	31	0.04	(0.03, 0.06)
Failure to gain weight or grow	56	0.06	(0.04, 0.07)	33	0.05	(0.03, 0.06)
Other abdominal condition	53	0.05	(0.04, 0.07)	27	0.04	(0.02, 0.05)
Kidney / bladder problems	24	0.02	(0.02, 0.03)	17	0.02	(0.01, 0.04)
Development problems with cheekbones	<5			<5		
Development problems with jaw	55	0.06	(0.04, 0.07)	71	0.10	(0.08, 0.12)
Development problems with tongue	21	0.02	(0.01, 0.03)	16	0.02	(0.01, 0.03)
Development problems with hands	15	0.02	(0.01, 0.02)	12	0.02	(0.01, 0.03)
Other development condition	50	0.05	(0.04, 0.07)	28	0.04	(0.03, 0.05)
Combined categories						
Development problems with eyes (including difficulties with vision or blindness)	98	0.10	(0.08, 0.12)	105	0.15	(0.12, 0.17)

Development problems with ears (including hearing loss or impairment)	284	0.29	(0.26, 0.32)	196	0.27	(0.24, 0.31)
Development problems with spine (including spine conditions)	12	0.01	(0.01, 0.02)	12	0.02	(0.01, 0.03)
Development problems with feet (including Talipes)	45	0.05	(0.03, 0.06)	32	0.05	(0.03, 0.06)
Biological sex specific conditions		***n=983***				
Hypospadias (males only n=983)	15	0.02	(0.01, 0.02)	-	-	-

[a]95% confidence intervals refer to the estimate of the prevalence within the population of interest; <5 at least one cell is disclosive by count or deduction

Table S3– Common syndromes associated with cleft lip and palate and the anomalies seen in participants of the Cleft Collective

Syndrome or sequence	Structural and functional anomalies reported in the literature	No. of participants diagnosed within sample	Structural and functional anomalies present in >10% / >20%[a] of diagnosed participants	No. of non-syndromic participants presenting two or more of the structural and functional anomalies that presented in those with the known syndrome/sequence (denominator n=1353)
Stickler syndrome<br>*Robin et al (2021)*[1]	Ocular difficulties, hearing loss, cleft palate (potentially associated with Robin sequence)[b] [2, 3], skeletal anomalies and early onset arthritis.	19	Developmental delay (n<5)<br>Heart condition (n<5)<br>Skeletal condition (n<5)<br>Other skin / musculoskeletal condition not included above (n<5)<br>Severe or persistent vomiting (n<5)<br>Failure to gain weight or grow (n<5)<br>Development problem with jaw (47.4%, n=9)[c]<br>Development problem with tongue (n<5)[c]<br>Other development condition (n<5)<br>Development problems with eyes (including difficulties with vision / blindness) (79.0%, n=15)<br>Development problems with ears (including hearing loss or impairment) (42.1%, n=8)<br>Development problems with spine (including spine conditions) (n<5)	184 (13.6%)

Van der Woude<br>*Lam et al (2010)*[4]<br>*Rizos et al (2004)*[5]	Cleft lip and/or palate with lower lip fistulae. Associated with high prevalence of hypodontia.	18	Other skin / musculoskeletal condition not included above (n<5)<br>Development problems with ears (including hearing loss or impairment) (33.3%, n=6)	16 (1.2%)
22q11 deletion<br>*McDonald-McGinn et al (2021)*[6]	Submucous cleft palate, bifid uvula or cleft palate. Congenital heart disease, immune deficiency, learning difficulties, distinctive facial characteristics and hearing loss. Anomalies of the eyes, larynx, gastrointestinal system, central nervous system, urinary and genital organs and skeletal system.	11	Developmental delay (63.6%, n=7)<br>Heart condition (54.6%, n=6)<br>Immune deficiency (n<5)<br>Other problems with heart / lungs / immune system (45.5%, n=5)<br>Other skin / musculoskeletal condition not included above (n<5)<br>Thyroid condition (n<5)<br>Severe / persistent gut abnormalities (n<5)<br>Other development condition (n<5)<br>Development problems with eyes (including difficulties with vision / blindness) (n<5)<br>Development problems with ears (including hearing loss or impairment) (n<5)<br>Development problems with spine (including spine conditions) (n<5)<br>Development problems with feet (including Talipes) (n<5)	182 (13.5%)
Craniosynostosis<br>*Kajdic et al (2018)*[7]	Facial and skull malformations, developmental delay. Ocular and respiratory anomalies.	9	Epilepsy / fits / convulsions (n<5)<br>Developmental delay (67.0%, n=6)<br>Other problems with heart / lungs / immune system (n<5)<br>Skeletal condition (n<5)	202 (14.9%)

			Other skin / musculoskeletal condition not included above (n<5)<br>Failure to gain weight or grow (n<5)<br>Development problems with eyes (including difficulties with vision / blindness) (n<5)<br>Development problems with ears (including hearing loss or impairment) (n<5)<br>Development problems with feet (including Talipes) (n<5)
CHARGE<br>*Hsu et al (2014)*[8]	Facial malformations and orofacial cleft. Blocking or narrowing of the nasal passage. Developmental delay. Immune deficiencies. Ocular anomalies (specifically coloboma), renal anomalies and anomalies of the cranial nerve, ears, hands and limbs. Genital hypoplasia. Malformation of the cardiovascular system. Faltering growth.	6	Developmental delay (83.3%, n=5)<br>Heart condition (n<5)<br>Other skin / musculoskeletal condition not included above (n<5)<br>Failure to gain weight or grow (n<5)<br>Other abdominal condition (n<5)<br>Development problems with eyes (including difficulties with vision / blindness) (100.0%, n=6)<br>Development problems with ears (including hearing loss or impairment) (100.0%, n=6)

[a]Where the number of participants with a given syndrome was greater than 10, structural and functional anomalies present in more than 10% of cases were detailed. Where the number of participants with a given syndrome was 10 or less, structural and functional anomalies present in more than 20% of cases were detailed.

[b]An orofacial cleft is associated with this syndrome / sequence but is not always present in a clinical population. Data for this study was obtained from The Cleft Collective, a longitudinal cohort study of children with an orofacial cleft and their families. Therefore, an orofacial cleft will be present in 100% of our sample.

[c]Based on the literature, likely to be associated with an additional syndrome.

[1] N. Robin, R. Moran, and L. Ala-kokko, "Stickler Syndrome," Adam MP, Mirzaa GM, Pagon RA, et al., editors. GeneReviews® [Internet].

[2] A. Giudice *et al.*, "Pierre Robin sequence: A comprehensive narrative review of the literature over time," *J. Stomatol. Oral Maxillofac. Surg.*, vol. 119, no. 5, pp. 419–428, 2018, doi: 10.1016/j.jormas.2018.05.002.

[3] A. Davies *et al.*, "Syndromes associated with Robin sequence: a national prospective cohort study," *Arch. Dis. Child.*, vol. 108, no. 1, pp. 42–46, 2022, doi: 10.1136/archdischild-2022-324722.

[4] A. K. Lam, D. J. David, G. C. Townsend, and P. J. Anderson, "Van der Woude syndrome: Dentofacial features and implications for clinical practice," *Aust. Dent. J.*, vol. 55, no. 1, pp. 51–58, 2010, doi: 10.1111/j.1834-7819.2009.01178.x.

[5] M. Rizos and M. N. Spyropoulos, "Van der Woude syndrome: A review. Cardinal signs, epidemiology, associated features, differential diagnosis, expressivity, genetic counselling and treatment," *Eur. J. Orthod.*, vol. 26, no. 1, pp. 17–24, 2004, doi: 10.1093/ejo/26.1.17.

[6] D. M. McDonald-McGinn, H. S. Hain, B. S. Emanuel, and E. H. Zackai, "22q11 . 2 Deletion Syndrome Summary Genetic counseling GeneReview Scope Diagnosis Suggestive Findings," *GeneReviews®*, pp. 1–24, 2020.

[7] N. Kajdic, P. Spazzapan, and T. Velnar, "Craniosynostosis-recognition, clinical characteristics, and treatment," *Bosn. J. Basic Med. Sci.*, vol. 18, no. 2, pp. 110–116, 2018, doi: 10.17305/bjbms.2017.2083.

[8] P. Hsu *et al.*, "CHARGE syndrome: A review," *J. Paediatr. Child Health*, vol. 50, no. 7, pp. 504–511, 2014, doi: 10.1111/jpc.12497.

Table S4 – Prevalence of parent reported diagnosed syndromes and structural and functional anomalies between ages 18 months and 5 years

Timepoint of parental questionnaire (n=370)[a]	Prevalence of syndrome and/or sequence	Prevalence of having 2 or more structural and functional anomalies
18 months	17.6% (n=65)	20.5% (n=76)
3 years	18.7% (n=69)	23.0% (n=85)
5 years	20.8% (n=77)	23.8% (n=88)

[a] Number of families who returned at least one questionnaire at all three timepoints
